# Longitudinal assessment of sleep and fatigue according to baby feeding method in postpartum women: a prospective observational study

**DOI:** 10.1186/s12884-024-06671-0

**Published:** 2024-08-12

**Authors:** An Mariman, Ignace Hanoulle, Dirk Pevernagie, Sarah-Jane Maertens, Isabelle Dehaene, Els Tobback, Liesbeth Delesie, Anne Loccufier, Ann Van Holsbeeck, Lara Moons, Dirk Vogelaers

**Affiliations:** 1 Centre for Integrative Medicine, Department of Physical Medicine and Rehabilitation, Ghent University Hospital, Corneel Heymanslaan 10, Ghent, B-9000 Belgium; 2 Faculty of Medicine and Health Sciences, Ghent University, Corneel Heymanslaan 10, Ghent, B-9000 Belgium; 3 Department of Pulmonary Medicine, Ghent University Hospital, Corneel Heymanslaan 10, Ghent, B-9000 Belgium; 4 Department of Gynecology and Obstetrics, Ghent University Hospital, Corneel Heymanslaan 10, Ghent, B-9000 Belgium; 5 Department of General Internal Medicine, Ghent University Hospital, Corneel Heymanslaan 10, Ghent, B-9000 Belgium; 6 Department of Gynecology, Saint-John Hospital Bruges, Ruddershove 10, Bruges, B-8000 Belgium; 7 Department of General Internal Medicine and Infectious Diseases, AZ Delta, Deltalaan 1, Roeselare, B-8800 Belgium; 8 Centre for Integrative Medicine, Ghent University Hospital, Corneel Heymanslaan 10, Ghent, B-9000 Belgium

**Keywords:** Feeding methods, Fatigue, Depression, Sleep quality, Choice behavior

## Abstract

**Background:**

Poor subjective sleep quality, depressive symptoms and fatigue occur frequently in postpartum. However, the dynamics of their respective associations from prepartum throughout the maternity period in function of baby feeding method have not been fully elucidated.

**Methods:**

Prospective, longitudinal study using validated questionnaires probing for sleep quality, insomnia, fatigue and depressive symptoms at 35–37 weeks of gestation and at 2, 8 and 12 weeks postpartum in the obstetric departments of two Flemish hospitals. Somers’d ordinal correlation was used for correlations between the results of questionnaires (ratio variables) and the feeding method variable (an ordinal variable); T tests (normal data) or Mann Whitney (non normal data) tests for equality of means; ordinal regression (‘Proportional odds model’) to investigate the predictive value of parameters at one moment on the feeding method choice at a later moment; logistic regression to investigate the predictive value of parameters on later change of feeding method.

**Results:**

188 women indicating a choice for either bottle or breastfeeding in prepartum (27–35 weeks’ gestation) were included. Higher fatigue assessed through the Fatigue Severity Scale within late pregnancy was moderately associated with primary bottle feeding choice. Fatigue decreased at early and late postpartum in bottle feeding (-0.38 ± 1.04; *p* = .110 and − 0.31 ± 1.01; *p* = .642 respectively), but remained unchanged from late pregnancy through early and late postpartum in breastfeeding (0.04 ± 1.21; *p* = .110 and − 0.27 ± 0.96; *p* = .642 respectively), resulting in similar fatigue in both feeding methods in early through late postpartum. There were no differences in sleep quality or insomnia symptoms at all time points. Presence of postpartum depressive symptoms were associated with early switching to bottle feeding (Somers’ d correlation 0.11 (*p* = .021).

**Conclusions:**

Fatigue and depressive symptoms are inversely associated with breastfeeding initiation or maintenance and influence feeding method dynamics.

**Supplementary Information:**

The online version contains supplementary material available at 10.1186/s12884-024-06671-0.

## Background

For several decades Western women rank fatigue in the top 5 postpartum health concerns [1]. The first six weeks postpartum represent the most vulnerable period. Different contributing factors include decreased total sleep time, less support by family and friends, increase in depressive symptoms, difficult children, postpartum blood loss, caesarean section and breastfeeding [2]. Surprisingly, in a recent systematic review only three studies could be identified exploring the relationship between infant feeding and maternal sleep patterns, requiring further analysis of this interaction [3].

Postpartum women report less sleep in the first weeks after delivery as compared to the gestational period [4], partly due to nighttime feeding and/or baby care as well as irregular baby sleep patterns [5]. Several studies report decreased total sleep time, sleep efficiency and increased wake after sleep onset [6–11]. Disturbed and/or low quality nighttime rest can lead to mother fatigue, inducing an increased risk of depressive symptoms and postpartum depression (PPD) [4, 12]. Strategies to avoid fatigue hence seem indicated [13].

In general, women have a higher risk for developing mood disorders, with a postpartum peak and a more than two-fold risk of needing mental health care during the first 3 months after delivery as compared to a year later [14]. Seemingly obvious underlying causes such as parity, civil state, relationship satisfaction and a previous history of depressive disorder have not shown significant relationships with the occurrence of PPD. Sleep disturbances however did impact significantly [15].

Breastfeeding is promoted in many health campaigns, as it is associated with significant reductions in child morbidity and mortality [16]. Hence, the World Health Organization aims at increasing the rate of exclusive breastfeeding for the first 6 months up to at least 50% by 2025 [17]. This is counteracted by a prevailing opinion that breastfeeding causes decreased sleep and/or sleep fragmentation, resulting in higher levels of fatigue and depressive feelings, leading to breastfeeding cessation [18, 19]. Health care workers experience a seeming conflict between promotion of breastfeeding and reduction of postpartum fatigue [20]. However, studies on effects of breast- versus bottle feeding on postpartum fatigue have been inconclusive [19].

In an earlier cross-sectional study on postpartum subjective sleep and fatigue, breastfeeding was associated with a worsening of sleep efficiency, compensated by a better sleep quality, and hence resulted in a similar global fatigue [21]. There were methodological limitations due to the cross-sectional design with a single postpartum time point studied.

We aimed at comparing subjective sleep quality, depressive symptoms and fatigue and their respective associations, from prepartum to early versus late postpartum in bottle versus breastfeeding mothers.

## Methods

### Design and setting of the study

Prospective longitudinal study in the obstetric departments of two Flemish hospitals, including four assessment points: time 0 (T0) between 35 and 37 weeks of gestation; time 1 (T1) in early postpartum, namely at 2 weeks after delivery; time 2 (T2) in late, i.e. at 8 weeks and time 3 (T3) at 6 months postpartum.

### Aims of the study

To investigate (formulated as research questions):

1. whether T0 parameters have predictive value for choice of feeding method on T1.
2. whether the feeding method is associated with the evolution between T0 and T1 of each individual subjective physical condition parameter.
3. whether the feeding method is associated with each individual physical condition parameter on T1.
4. which T1 parameters predict a switch from breast- or mixed to either mixed or mere bottle feeding at T2.
5. the evolution of subjective physical condition parameters after T1 in the 3 feeding method groups.

### Participants

Participants were recruited between 27 and 35 weeks of singleton pregnancy within regular consultations in the obstetric departments of two different hospitals between 1.1.2017 and 1.8.2019. Researchers were regularly present to recruit participants and obtain informed consent.

Women who indicated that they would exclusively choose for either breast- or bottle feeding were eligible for being recruited for the study. They needed to be at least 18 years old and fluent in Dutch as mother tongue. Mothers were excluded if their infant remained hospitalized in the neonatal intensive care unit. After recruitment the choice of mixed, breast- or bottle feeding was allowed. Subjects preferring mixed feeding were included as a third group in order to assess the dynamics of feeding method.

### Data collection tools

Sociodemographic data and maternal and infant-related factors with possible influence on sleep and/or fatigue (organic conditions in the infant, pre-existing chronic fatigue syndrome or psychiatric disorders and use of psychotropic drugs) were collected using a study-specific self-administered questionnaire. Validated questionnaires at the different time points included the Pittsburgh Sleep Quality Index (PSQI) [22], the Insomnia Severity Index (ISI) [23], the Fatigue Severity Scale (FSS) [24] and the Center for Epidemiologic Studies Depression Scale (CES-D) [25, 26], probing the dimensions of sleep quality, insomnia, fatigue and depressive symptoms, respectively. These questionnaires were offered online, with the possibility of a paper backup. If needed, appropriate reminders were sent.

### Statistical analyses

Similar to a previous study using the PSQI [4], to detect a standard effect size of 0.61 with a t-test comparing means, a total of at least 44 participants was required for equally-sized feeding groups, as identified after power calculation (α = 0.05, β = 0.20, standardized effect size Cohen’s d = 0.61). For two groups with a different number of participants in each feeding group, the harmonic mean of the two numbers: N’= (2*N_A_*N_B_)/( N_A_+ N_B_) gives the equivalent number of participants in equally-sized groups and serves as a basis for power calculations.

Descriptive statistics were calculated by using means and standard deviations for continuous variables and frequencies for categorical variables. For the first question, we used Somers’d ordinal correlations and ordinal regressions (‘cumulative’ or ‘proportional’ odds model) with the feeding method variable on T1 as dependent variable. The test of parallel lines assessed whether the relationships between the independent variables and the logits were the same for all the logits.

For the second question, involving bivariate relations between evolution of physical conditions with the feeding method, Somers’d ordinal correlations were used. To compare the means of the breastfeeding and the bottle feeding group, t-tests (in the cases where the two samples were normally distributed according to the Shapiro Wilk test of normality) and Mann Whitney tests (in the other cases) were used.

The third and the fifth question, equally involving bivariate relations between physical conditions and the feeding method, were similarly handled with Somers’d ordinal correlations, t-tests and Mann Whitney tests.

The fourth question resembles the first question and was handled with Somers’d ordinal correlations and logistic regression (the change of feeding method variable as a dependent variable).

All analyses were two-tailed, with the level of significance at *p* < .05.

Potential biases were addressed by performing analyses both on the per protocol samples of women remaining on the initial feeding method with additional analysis of the switchers on the one hand, as well as on the effective sample with the actual specific feeding method at the different time points. The cases with missing data were omitted (“complete case analyses/listwise deletion”).

## Results

### Dynamics of feeding subgroup composition

**Fig. 1:**
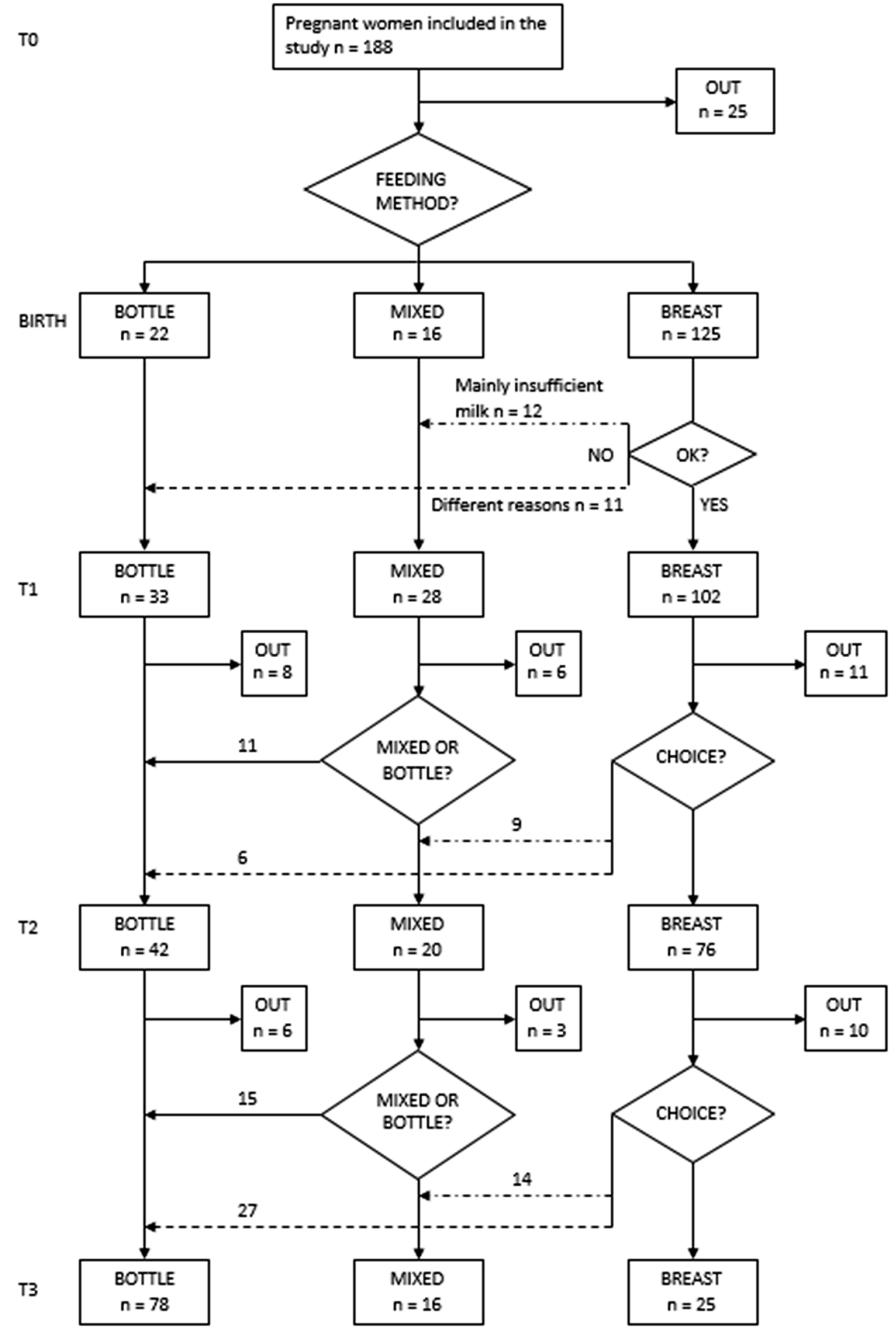
Flowchart of dynamics of choice of feeding method. Reasons for non-participation at each stage were not probed.; T1: early postpartum (2 weeks after delivery); T2: late postpartum (8 weeks after delivery); T3: 6 months postpartum

Out of 163 recruited subjects, 85% still participated at T2 vs. 73% at T3. Feeding choice dynamics, including mixed feeding and method switching, from early to late postpartum, is represented in a flow chart (Fig. 1). The harmonic means at each time point of follow up is given in Table 1. The predefined criteria for participation rates were met at T1 and at T2, but not at T3.

**Table 1:** Overview of the assessment time points and respective harmonic means

	Total	Exclusively breastfeeding	Mixed, breast- and bottle feeding	Exclusively bottle feeding	Harmonic mean*
T0: Prenatal period (35-37 weeks of gestation)	188				
T1: early postpartum (2 weeks after delivery)	163	102 (63%)	28 (17%)	33 (20%)	50
T2: late postpartum (8 weeks after delivery)	138	76 (55%)	20 (14%)	42 (30%)	54
T3: late postpartum (6 months after delivery)	119	25 (21%)	16 (13%)	78 (65%)	38

*required minimum of the power calculation: 44

### Demographics

Breastfeeding participants had significantly higher employment rates than bottle or mixed feeding subjects. They had more often anemia and their mean total sleep time before pregnancy was significantly lower than that of the other two groups. For all other characteristics, there were no significant differences between the three groups. Full socio-demographic characteristics and factors potentially influencing sleep and/or fatigue are shown in Appendix 1 (provided as online supplement).

### Longitudinal assessment of sleep, insomnia, fatigue and depression

The research questions were addressed using the descriptive longitudinal data reported in Table 2.

**Table 2:** Descriptives of PSQI, ISI, FSS, CESD on the 4 time points and their evolution between two moments

	Total sample	Breastfeeding at T1 (C1=0)	Mixed feeding at T1 (C1 = 1)	Bottle feeding at T1 (C1 = 2)	Correl.^(^*^)^ with C1 (*P* value)	*P* value of test ^(^†^)^ of equal means between breast- and bottle feeding
	*N* = 163	*N*= 102	*N* = 28	*N* = 33		
PSQI0: mean ± SD	6.97 ± 3.06	6.72 ± 2.93	7.36 ± 2.98	7.42 ± 3.52	0.06 (0.223)	0.341
PSQI1: mean ± SD	7.56 ± 3.02	7.44 ± 2.87	7.96 ± 3.06	7.58 ± 3.45	0.02 (0.734)	0.832
PSQI1 - PSQI0: mean ± SD	0.59 ± 3.90	0.73 ± 3.37	0.61 ± 3.91	0.15 ± 5.30	-0.01 (0.845)	0.721
ISI0: mean ± SD	8.73 ± 4.41	8.34 ± 4.37	9.18 ± 4.37	9.55 ± 4.58	0.08 (0.101)	0.142
ISI1: mean ± SD	8.40 ± 4.88	8.29 ± 5.03	8.96 ± 4.80	8.27 ± 4.58	0.02 (661)	0.854
ISI1 - ISI0: mean ± SD	-0.33 ± 5.59	-0.05 ± 5.86	-0.21 ± 4.17	-1.27 ± 5.81	-0.03 (0.501)	0.421
**FSS0: mean ± SD**	**3.76 ± 1.17**	**3.60 ± 1.15**	**3.88 ± 1.21**	**4.15 ± 1.11**	**0.11** ^**‡**^ **(0.023)**	**0.022** ^**‡**^
FSS1: mean ± SD	3.71 ± 1.19	3.66 ± 1.19	3.89 ± 1.24	3.72 ± 1.16	0.02 (0.624)	0.804
**FSS1 - FSS0: mean ± SD**	**-0.04 ± 1.19**	**0.07 ± 1.21**	**0.01 ± 1.21**	**-0.43 ± 1.06**	**-0.18** ^**‡**^ **(0.049)**	**0.041** ^**‡**^
CESD0: mean ± SD	18.37 ± 5.18	17.82 ± 4.87	20.14 ± 5.40	18.55 ± 5.71	0.05 (0.272)	0.753
CESD1: mean ± SD	18.99 ± 6.95	18.34 ± 6.98	21.00 ± 7.47	19.30 ± 6.19	0.07 (0.163)	0.412
CESD1 - CESD0: mean ± SD	0.63 ± 6.77	0.52 ± 6.90	0.86 ± 8.10	0.76 ± 5.12	0.01 (0.894)	0.862
	**Total sample**	**Breastfeeding at T2 (C2=0)**	**Mixed feeding at T2 (C2 = 1)**	**Bottle feeding at T2 (C2 = 2)**	Correl.(*) with C2 (P value)	**P value of test (†) of equal means between breast- and bottle feeding**
	***N*** = 142	***N***= 77	***N*** = 21	***N*** = 44		
PSQI2: mean ± SD	6.39 ± 3.22	6.32 ± 3.49	6.67 ± 2.50	6.36 ± 3.10	0.02 (0.794)	0.951
PSQI2 - PSQI1: mean ± SD	-1.30 ± 3.08	-0.94 ± 2.91	-1.48 ± 3.04	-1.84 ± 3.37	-0.10 (0.164)	0.121
ISI2: mean ± SD	7.22 ± 5.07	6.96 ± 5.36	7.00 ± 3.76	7.79 ± 5.13	0.07 (0.334)	0.344
ISI2 - ISI1: mean ± SD	-1.17 ± 4.23	-0.99 ± 4.54	-1.19 ± 3.86	-1.49 ± 3.87	-0.04 (0.601)	0.542
FSS2: mean ± SD	3.51 ± 1.32	3.33 ± 1.35	3.56 ± 1.06	3.78 ± 1.34	0.12 (0.084)	0.083
FSS2 - FSS1: mean ± SD	- 0.25 ± 1.01	-0.27 ± 0.96	-0.37 ± 1.18	-0.15 ± 1.03	0.01 (0.874)	0.742
CESD2: mean ± SD	18.48 ± 8.00	17.74 ± 7.46	17.48 ± 5.47	20.28 ± 9.66	0.08 (0.271)	0.211
CESD2 - CESD1: mean ± SD	-0.18 ± 7.46	-0.32 ± 6.95	-2.48 ± 7.61	0.02 ± 8.21	-0.05 (0.462)	0.604
	**Total sample**	**Breastfeeding at T3 (C3=0)**	**Mixed feeding at T3 (C3 = 1)**	**Bottle feeding at T3 (C3 = 2)**	Correl.(*) with C3 (P value)	**P value of test (†) of equal means between breast- and bottle feeding**
	***N*** = 119	***N***= 25	***N*** = 16	***N*** = 78		
PSQI3: mean ± SD	5.46 ± 3.15	5.68 ± 2.85	4.56 ± 2.37	5.47 ± 2.94	-0.02 (0.761)	0.481
PSQI3 - PSQI2: mean ± SD	-0.76 ± 3.23	-0.24 ± 3.55	0.13 ± 2.25	-1.19 ± 2.91	-0.12 (0.094)	0.212
ISI3: mean ± SD	6.91 ± 5.04	7.16 ± 4.55	5.88 ± 4.49	7.18 ± 4.78	-0.01 (0.874)	0.594
ISI3 - ISI2: mean ± SD	0.03 ± 4.43	1.00 ± 5.03	1.69 ± 3.11	-0.59 ± 4.41	**-0.16** ^**‡**^ **(0.032)**	0.131
FSS3: mean ± SD	3.37 ± 1.30	3.54 ± 1.43	3.15 ± 1.52	3.36 ± 1.24	-0.01 (0.861)	0.602
**FFS3 - FFS2: mean ± SD**	**-0.10 ± 1.07**	**0.32 ± 1.07**	**0.39 ± 0.74**	**-0.34 ± 1.07**	**-0.23** ^**§**^ **(0.000)**	**0.009**
CESD3: mean ± SD	17.63 ± 7.29	17.04 ± 6.26	15.94 ± 4.89	17.60 ± 6.58	0.04 (0.621)	0.931
CESD3 - CESD2: mean ± SD	-0.23 ± 6.76	-0.08 ± 4.72	0.44 ± 5.27	-0.91 ± 6.21	-0.05 (0.504)	0.584

^(^*^)^ Somers’ d ordinal correlation

^(^†^)^ T test (in the cases where the two samples are normally distributed according to the Shapiro-Wilk test of normality) or Mann Whitney test (in the other cases)

Measurement moments: T0, 35-37 weeks pregnancy; T1, 2 weeks postpartum; T2, 8 weeks postpartum; T3, 6 months postpartum. PSQI0, PSQI1, PSQI2, PSQI3: PSQI on moments T0 respectively T1, T2, T3 and similarly for ISI, FSS, CESD

Ci is a variable indicating if the mother is breastfeeding, bottle feeding or giving mixed feeding on time point Ti

‡ *p* < .05, § *p* < .01

SD: Standard deviation

#### Initial choice of feeding method

Concerning the first research question, fatigue on T0 has predictive value for the feeding method choice on T1 (Somers’d correlations in Appendix 1 and Table 2, t-tests in Table 2 and ordinal regression in Table 3). The predictor variable is significant (*p* = .02) explaining 2% of the total variance. Other variables on T0 could not contribute significantly to the prediction of the feeding method. In particular mother’s age showed no correlation with initial feeding method.

**Table 3:** Ordinal regression (‘Proportional odds model’) with the feeding method variable C1 on measurement moment T1 (two weeks postpartum) as the dependent variable, and parameters on measurement T0 (35 weeks pregnancy) as independent variables

Coefficients of predictor variables	Estimate B (Standard Error)	Wald	df	Sig	95% Confidence Interval for B	
					Lower Bound	Upper Bound
FSS on T0 (values from finel regression)	0.33* (0.14)	5.70	1	0.02	0.06	0.61
Employment rate	-0.01 (0.01)	1.89	1	0.17	-0.02	0.00
Mean number of sleep hours before pregnancy	0.24 (0.18)	1.71	1	0.19	-0.12	0.60

** *p*<0,01, *p<0,05

*Notes*:

1. C1 = 0, breastfeeding; C1 = 1, mixed feeding; C1 = 2, bottle feeding

2. The test of parallel lines indicated that the assumption of ordinal regression, namely that the relationships between the independent variables and the logits are the same for all the logits, is plausible (sig. = 0.74)

3. With FSS on T0 already in the model, no other variables on T0 did have a significant contribution to the explaining of C1. The results are shown for the variables employment rate and mean number of sleep hours before pregnancy when FSS on T0 is in the model. The values of FSS on T0 are from the final regression, without any other variables in the model

4. Other results from the final model: Pseudo R-square = 0.02 (Mc Fadden, R²L); 0.04 (Cox and Snell); 0.04 (Nagelkerke)

Model fit: Chi-square = 5.94 (df = 1), Sig = 0.02

In relation to the second research question, the feeding method was correlated (Somers’ d correlation = − 0.18, *p* = .049) with the evolution of fatigue (Table 2, variable FSS1 – FSS0) between T0 and T1. A t-test showed significantly (*p* = .041) more improvement in fatigue in the bottle feeding as compared to the breastfeeding group.

No significant difference between the 3 feeding groups was found (Somers’d correlations and t-tests) for the evolution of the other subjective physical condition parameters (Table 2, the variables PSQI1 – PSQI0, ISI1 – ISI0, CESD1 – CESD0).

In relation to the third research question, no significant difference between the three groups was found (Somers’d correlations and t-tests) for the subjective physical condition parameters on T1 (Table 2, the variables PSQI1, ISI1, FSS1, CESD1).

#### Switch of feeding method

Only depression on T1 seems to influence mothers giving breastfeeding or mixed feeding on T1 to change their feeding method in the subsequent period (Appendix 2 in online supplement, summarizing statistical results in relation to the fourth research question and revealing the same pattern with Somers’d correlations and logistic regression). The predictor variable CESD1 explains 7.5% of the variance (*p* = .002). No other variable on T1 could contribute significantly to the prediction of the change of feeding method on T2.

#### Evolution of subjective physical condition parameters

The statistical results in connection with the fifth question, examining the evolution of the subjective physical condition parameters after T1 in the three feeding groups, are again shown in Table 3, in the second and third part of the table. The evolution of FSS (variable FSS3 – FSS2) between T2 and T3 was significantly different between the breast- and the bottle feeding groups (*p* = .000), as the bottle feeding group showed more improvement in fatigue than the breastfeeding group.

**Fig. 2:**
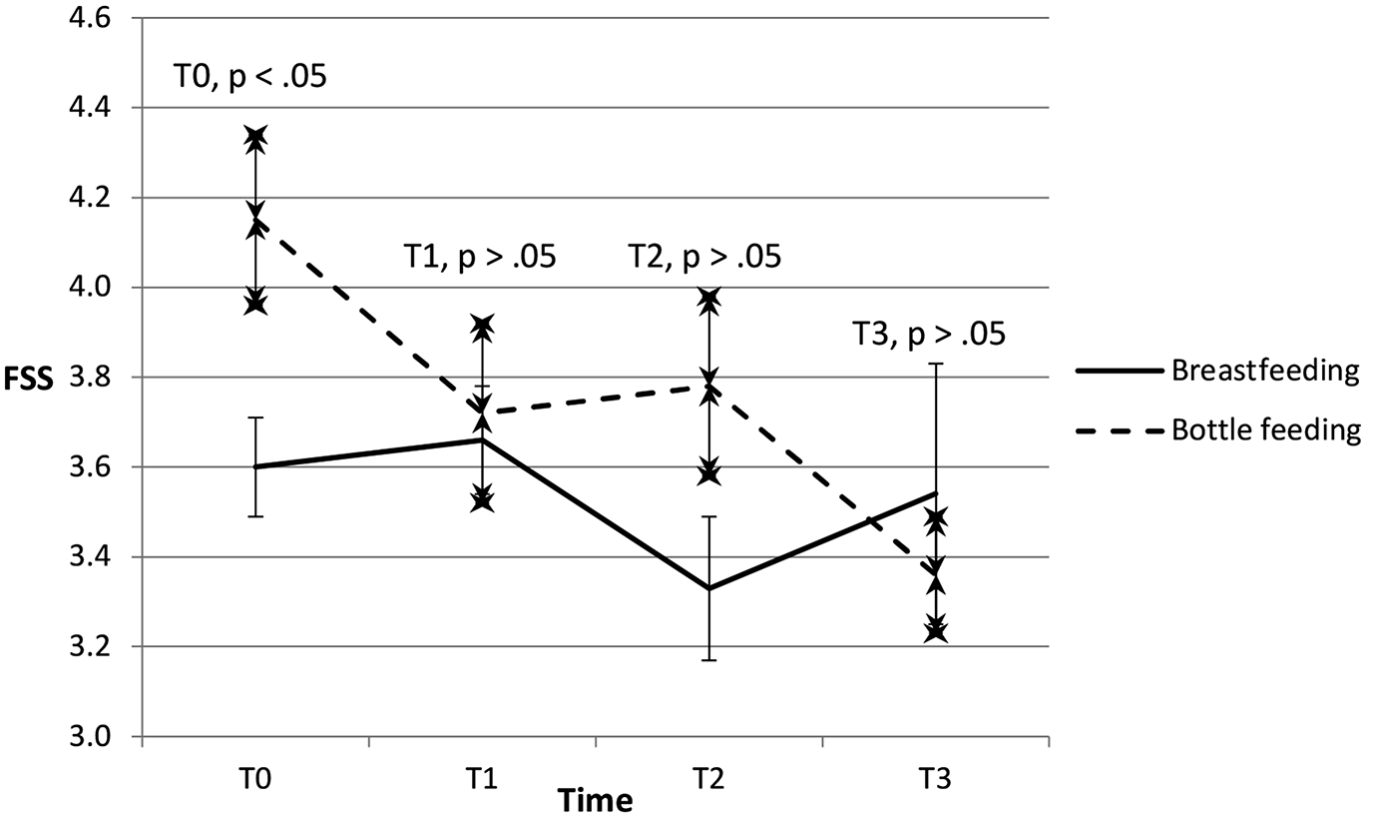
Evolution of fatigue from T0 to T3 for the breastfeeding and bottle feeding groups. Fatigue Severity Scale (FSS); values are mean ± standard error

**Fig. 3:**
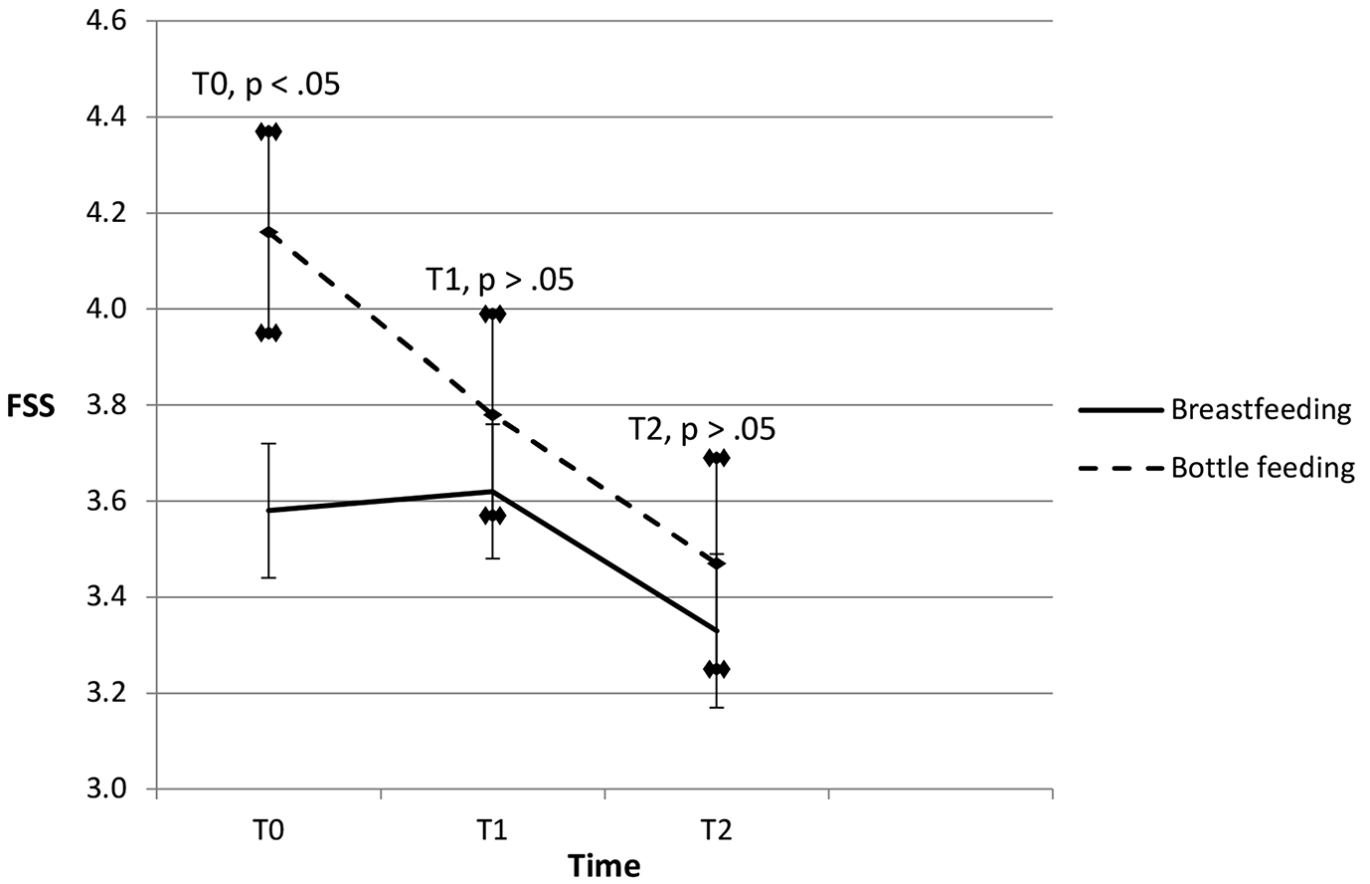
Evolution of fatigue for women who do not change their feeding method at T2. Fatigue Severity Scale (FSS); values are mean ± standard error

The evolution of fatigue is illustrated in Figs. 2 and 3 indicating a difference between the bottle and breastfeeding group. The bottle feeding group has more fatigue on T0, and descends to the same level as the other group on T1.

Mothers who continue with the same feeding method on T2 show the same descent in fatigue in both groups (Fig. 3). In contrast, Fig. 2 shows more fatigue on T2 in the bottle feeding group: some breastfeeding mothers on T1 change their feeding method and the tiredness of these now bottle feeding mothers on T2 explains the difference between the two figures. On T3, there is no residual difference in fatigue between the two groups. Descriptive data at T0, T1 and T2 of mothers who did not change versus those changing their feeding method at T2 respectively are reported in Appendix 3 and 4 (online supplement).

## Discussion

### Main findings

In this non-randomized, prospective, longitudinal study, a higher level of late pregnancy fatigue was moderately associated with a primary choice for bottle feeding. No significant difference in fatigue appeared between breast- and bottle feeding in both early and later postpartum. Fatigue decreased at early and late postpartum in bottle feeding, remaining unchanged from late pregnancy through early and late postpartum in breastfeeding. Sleep quality or insomnia symptoms were similar at all time points. Depressive symptoms in postpartum were associated with switching to bottle feeding.

The longitudinal approach, including scores in late pregnancy, revealed that late pregnancy fatigue was associated with a choice for bottle feeding. This late pregnancy fatigue only explained a minor part of the variance in feeding method decision. Subsequently, there was a continued improvement in fatigue in bottle feeding in early and late postpartum, in the order of -0.43 in FSS, similar to clinically relevant changes reported in other populations [27–30]. Fatigue remained unchanged in breastfeeding. Hence, there was no residual difference in fatigue between both feeding methods in early postpartum, as the trends converged.

### Interpretation

Fatigue is commonly experienced in postpartum, particularly in primiparae [31]. There is a general perception that fatigue is more likely linked to breastfeeding. However, perceived fatigue scores at three time points (2–4 days, 6 and 12 weeks postpartum) were not significantly different for all feeding methods [19], in line with the cross-sectional analysis of consecutive postpartum time points in the present study. However, the dynamics of feeding choice in late pregnancy seem to play an underestimated role. In the present study the choice for bottle feeding in the more fatigued women at T1 and the subsequent switch from breast- to bottle feeding between early and late postpartum due to depressive symptoms should be viewed as real life phenomena influencing group composition. Due to these presumed selective processes, cross-sectional studies may fail to reveal real differences.

Fatigue may also be related with insomnia and depression. Increased rumination and insomnia are common in pregnancy and are associated with depression and suicidal ideation [32]. In the present study, depressive symptoms or insomnia, although frequent in late pregnancy, did not influence the initial choice of feeding method. However, depressive symptoms at early postpartum were associated with an earlier switch to bottle feeding. In several studies, pregnancy associated depression predicted shorter breastfeeding duration but not intention or initiation [33]. In a Brazilian cross-sectional study [34], PPD reduced exclusive breastfeeding. In a large Australian prospective study in primiparae, women reporting depressive symptoms at 3 months postpartum had significantly lower rates of breastfeeding at 6 months postpartum (49% vs. 61%) [35]. Early postnatal depressive symptoms appear a significant driver in decisions to cease breastfeeding. However, in a Norwegian prospective study, depressive symptoms during the last trimester, or at 4 and 6 months postpartum, did not impact on breastfeeding behaviour at any of the postpartum time points [36]. Comparison with the present study may be hampered by methodological issues, mainly due to additional recruitment at different time points in the postpartum period instead of relying on a strictly prospective protocol.

Sleep quality, insomnia severity and frequency of depressive symptoms were not significantly different at baseline and did not diverge in early or in late postpartum. Yet, the incidence of clinical insomnia and poor subjective sleep quality is high in both groups, as pointed out by Kalmbach et al. [32].

In two different cross-sectional studies subjective sleep quality and fatigue were compared in a time frame between 4 and 14 or 16 weeks postpartum respectively [21, 37] using global PSQI and the CIS as a measure of daytime functioning. In both studies global PSQI (but not PSQI components) and fatigue were similar. There was a significant positive correlation between total sleep quality and fatigue levels. Postpartum sleep changes and fatigue may have a similar association with mood disturbances, regardless of the feeding method. In Dørheim et al. postnatal women with depressive symptoms reported poorer subjective sleep with significantly higher PSQI scores 2 months after delivery as compared with non-depressed women [4]. The empirical, reasonable assumption that sleep disturbance during the perinatal period is significantly associated with an increased risk of depression [12] could not be substantiated in the present study. This longitudinal study highlights that fatigue in late pregnancy and depressive symptoms in early postpartum need to be taken into consideration, as they could have been partial drivers for initial choice for and switch to bottle feeding, possibly leading to subsequent fatigue improvement.

From this study it becomes clear that awareness for fatigue and depressive symptoms during pregnancy is important. In order to conserve the multiple recognized advantages of breastfeeding, it should be advocated to screen systematically at different time points throughout late pregnancy and postpartum for daytime functioning, fatigue, depressive symptoms and sleep quality and to act preemptively. Future mothers should be warned of the potential for postpartum fatigue and encouraged to organize means for assistance through this potentially difficult period [19]. Timely targeted interventions such as cognitive-behavioral therapy for insomnia can reduce depressive symptoms, increase the level of activity and motivation and significantly improve subjective sleep parameters and daytime fatigue, both physically and mentally [38]. While the impact on feeding method was not reported, a systematic review including 15 studies has shown that non-pharmacological sleep interventions may improve subjective maternal sleep quality [39]. Education on the misperception of breastfeeding contributing to fatigue in itself may avoid premature discontinuation [20].

### Strengths and limitations

The longitudinal design of the present study overcomes the limitations of cross-sectional investigations comparing bottle and breastfeeding at a single time point. In difference to a randomized controlled trial, this real life and hence generalizable study considered the complex dynamics of switching from breast- to either bottle or mixed feeding. The high participation rate in early postpartum reflects participant motivation but could not be achieved in late postpartum. The lack of data on parity is an obvious limitation. Future research should include larger sample sizes and be more continuous throughout both pre- and postpartum, including day to day diary parameters assessing intra- and inter-individual variability. Other demographic characteristics of likely relevance, such as maintaining work in late pregnancy and timing of return to work, should be included.

## Conclusions

This cohort study demonstrated the role of fatigue in late pregnancy towards a primary choice of bottle feeding and of depressive symptoms in early postpartum in switching away from the initial choice for breastfeeding. Enhanced screening for fatigue and depressive symptoms may hence be warranted when aiming at high levels of breastfeeding, and should be integrated in the practice of health care workers involved as well as into the education of women.

### Electronic supplementary material

Below is the link to the electronic supplementary material.

Supplementary Material 1

Supplementary Material 2

Supplementary Material 3

Supplementary Material 4

## Data Availability

Data cannot be shared publicly because the hospital policy restricts secondary use of the data without an advice of the Data Access Committee. Data are available upon reasonable request made to the Data Access Comité of the University Hospital of Ghent.

## Abbreviations

FSS: Fatigue Severity Scale
PPD: Postpartum depression
PSQI: Pittsburgh Sleep Quality Index
CES-D: Center for Epidemiologic Studies Depression Scale

## References

[1] Troy N, Dalgas-Pelish P. The development of a self-care guide for the management of postpartum fatigue. Appl Nurs Res. 1995;8:92–6. 7598523 10.1016/S0897-1897(95)80550-8

[2] Milligan R, Parks P, Kitzman H, Lenz ER. Measuring women’s fatigue during the postpartum period. J Nurs Meas. 1997;5:3–16. 9505466 10.1891/1061-3749.5.1.3

[3] Witkowska-Zimny M, Zhyvotovska A, Isakov R, Boiko DI, Nieradko-Iwanicka B. Maternal sleeping problems before and after childbirth - A systematic review. Int J Womens Health. 2024;16:345–71. 38455339 10.2147/IJWH.S446490 PMC10918694

[4] Dørheim S, Bondevik G, Eberhard-Gran M, Bjorvatn B. Sleep and depression in postpartum women: a population-based study. Sleep. 2009;32(7):847–55. 19639747 10.1093/sleep/32.7.847 PMC2704916

[5] Hunter L, Rychnovsky J, Yount S. A selective review of maternal sleep characteristics in the Postpartum Period. J Obstetric Gynecologic Neonatal Nurs. 2009;38:60–8. 10.1111/j.1552-6909.2008.00309.x 19208049

[6] Rychnovsky J, Hunter L. The relationship between sleep characteristics and fatigue in healthy postpartum women. Women’s Health Issues. 2009;19:38–44. 19111786 10.1016/j.whi.2008.07.015

[7] Matsumoto K, Shinkoda H, Kang M, Seo Y. Longitudinal study of mother’s sleep-wake behaviors and circadian time patterns from late pregnancy to postpartum-monitoring of wrist actigraphy and sleep logs. Biol Rhythm Res. 2003;34:265–78. 10.1076/brhm.34.3.265.18812

[8] Gay CL, Lee KA, Yee S. Sleep patterns and fatigue in new mothers and fathers. Biol Res Nurs. 2005;5:311–8. 10.1177/1099800403262142 PMC1307172 15068660

[9] Signal TL, Gander PH, Dangalli NR, Travier N, Firestone RT, Tuohy JF. Sleep duration and quality in healthy nulliparous and multiparous women across pregnancy and post-partum. Australian New Z J Obstet Gynecol. 2007;47:16–22. 10.1111/j.1479-828X.2006.00672.x 17261094

[10] Bey B, Milgrom J, Ericksen J, Trinder J. Subjective perception of sleep but not its objective quality is associated with immediate postpartum mood disturbances in healthy women. Sleep. 2010;33:531–8. 20394323 10.1093/sleep/33.4.531 PMC2849793

[11] Doan T, Gay CL, Kennedy HP, Newman J, Lee KA. Nighttime breastfeeding behavior is associated with more nocturnal sleep among first-time mothers at one month postpartum. J Clin Sleep Med. 2014;10:313–9. 24634630 10.5664/jcsm.3538 PMC3927438

[12] Okun ML. Disturbed sleep and Postpartum Depression. Curr Psychiatry Rep. 2016;18:66. 27222140 10.1007/s11920-016-0705-2

[13] Taylor J, Johnson M. How women manage fatigue after childbirth. Midwifery. 2010;26:367–75. 18771828 10.1016/j.midw.2008.07.004

[14] Musk-Olsen T, Laursen TM, Pedersen CB, Mors O, Mortensen PB. New parents and mental disorders: a population-based registry study. JAMA. 2006;296:2582–89. 17148723 10.1001/jama.296.21.2582

[15] Goyal D, Gay C, Lee K. Fragmented maternal sleep is more strongly correlated with depressive symptoms than infant temperament at three months postpartum. Arch Women Ment Health. 2009;12:229–37. 10.1007/s00737-009-0070-9 PMC2700868 19396527

[16] Mammas IN, Greenough A, Theodoridou M, Kramvis A, Rusan M, Melidou A, Korovessi P, Papaioannou G, Papatheodoropoulou A, Koutsaftiki C, Liston M, Sourvinos G, Spandidos DA. Paediatric Virology and its interaction between basic science and clinical practice (review). Int J Mol Med. 2018;41:1165–76. 29328393 10.3892/ijmm.2018.3364 PMC5819919

[17] World Health Organisation. Breastfeeding. [Internet]. https://www.who.int/health-topics/breastfeeding#tab=tab_2. [Accessed 2022].

[18] Galbally M, Lewis A, McEgan K, Scalzo K, Islam A. Breastfeeding and infant sleep patterns: an Australian population study. J Pediatr Child Health. 2013;49:e147–52. 10.1111/jpc.12089 23331519

[19] Callahan S, Séjourné N, Denis A. Fatigue and breastfeeding: an inevitable partnership? J Hum Lactation. 2006;22(2):182–7. 10.1177/0890334406286972 16684906

[20] Cloherty M, Alexander J, Holloway I. Supplementing breast-fed babies in the UK to protect their mothers from tiredness or distress. Midwifery. 2004;20:94–201. 15177864 10.1016/j.midw.2003.09.002

[21] Tobback E, Behaeghel K, Hanoulle I, Delesie L, Loccufier A, Van Holsbeeck A, Vogelaers D, Mariman A. Comparison of subjective sleep and fatigue in breast- and bottlefeeding mothers. Midwifery. 2017;47:22–7. 28232215 10.1016/j.midw.2017.01.009

[22] Buysse DJ, Reynolds CF, Monk TH, Berman SR, Kupfer DJ. The Pittsburgh Sleep Quality Index: a new instrument for psychiatric practice and research. Psychiatry Res. 1989;28:193–213. 2748771 10.1016/0165-1781(89)90047-4

[23] Morin CM, Belleville G, Bélanger L, Ivers H. The insomnia severity index: psychometric indicators to detect insomnia cases and evaluate treatment response. Sleep. 2011;34(5):601–8. 21532953 10.1093/sleep/34.5.601 PMC3079939

[24] Krupp LB, La Rocca MG, Muir-Nash J, Steinberg AD. The fatigue severity scale: application to patients with multiple sclerosis and systemic lupus erythematosus. Arch Neurol. 1989;46:1121–3. 2803071 10.1001/archneur.1989.00520460115022

[25] Callahan LF, Kaplan MR, Pincus T. The Beck Depression Inventory, Center for Epidemiological Studies Depression Scale (CES-D) and general well-being schedule depression subscale in rheumatoid arthritis. Criterion contamination of responses. Arthritis Care Res. 1991;4:3–11. 11188584 10.1002/art.1790040103

[26] Radloff L. A self-report depression scale for research in the general population. J Appl Psychol Measurements. 1977;1:385–401. 10.1177/014662167700100306

[27] Valko PO, Bassetti CL, Bloch KE, Held U, Baumann CR. Validation of the fatigue severity scale in a Swiss cohort. Sleep. 2008;31(11):1601–7. 19014080 10.1093/sleep/31.11.1601 PMC2579971

[28] Rosa K, Fu M, Gilles L, Cerri K, Peeters M, Bubb J, Scott J. Validation of the fatigue severity scale in Chronic Hepatitis C. Health Qual Life Outcomes. 2014;12:90. 24915781 10.1186/1477-7525-12-90 PMC4094687

[29] Goligher EC, Pouchot J, Brant R, Kherani RB, Avina-Zubietta AJ, Lacaille D, Lehman AJ, Ensworth S, Kopec J, Esdaile JM, Liang MH. Minimal clinically important difference for 7 measures of fatigue in patients with systemic lupus erythematosus. J Rheumatol. 2008;35:635–42. 18322987

[30] Pouchot J, Kherani RB, Brant R, Lacaille D, Lehman AJ, Ensworth S, Kopec J, Esdaille JM, Liand MH. Determination of the minimal clinically important difference for seven fatigue measures in rheumatoid arthritis. J Clin Epidemiol. 2008;61(7):705–13. 18359189 10.1016/j.jclinepi.2007.08.016 PMC2486378

[31] Henderson J, Alderdice F, Redschaw M. Factors associated with maternal postpartum fatigue: an observational study. BMJ Open. 2019;9e025927. 10.1136/bmjopen-2018-025927. 10.1136/bmjopen-2018-025927 PMC6661702 31352411

[32] Kalmbach DA, Cheng P, Ong JC, Ciesla JA, Kingsberg SA, Shanga R, Swanson LM, O’Brien LM, Roth T, Drake CL. Depression an suicidal ideation in pregnancy: exploring relationships with insomnia, short sleep, and nocturnal rumination. Sleep Med. 2020;65:62–73. 31710876 10.1016/j.sleep.2019.07.010 PMC6980654

[33] Diaz CC, Figueiredo B. Breastfeeding and depression: a systematic review of the literature. J Affect Disord. 2015;171:142–54. 25305429 10.1016/j.jad.2014.09.022

[34] Silva CS, Lima MC, Sequeira-de-Andrade LAS, Oliveira JS, Monteiro JS, Lima NMS, Santos RMAB, Lira PIC. Association between postpartum depression and the practice of exclusive breastfeeding in the first three months of life. J Pediatr (Rio J). 2017;93(4):356–64. 28034730 10.1016/j.jped.2016.08.005

[35] Woodhouse H, James J, Gartland D, McDonald E, Brown SJ. Maternal depressive symptoms at three months postpartum and breastfeeding rates at six months postpartum: implications for primary care in a prospective cohort study of primiparous women in Australia. Women Birth. 2016;29:381–7. 27450375 10.1016/j.wombi.2016.05.008

[36] Haga SM, Lisoy C, Drozd F, Valla L, Slinning K. A population-based study of the relationship between perinatal depressive symptoms and breastfeeding: a cross-lagged panel study. Arch Womens Ment Health. 2018;21:235–42. 29063201 10.1007/s00737-017-0792-z

[37] Atas AN, Ozerdogan N. Perceived sleep quality and fatigue in a Population of New Mothers: a cross-sectional study comparing relationships with Breastfeeding and Bottle-feeding. Breastfeed Med. 2021 Sep;13. 10.1089/bfm.2021.0040. 10.1089/bfm.2021.0040 34516778

[38] Swanson LM, Flynn H, Adams-Mundy JD, Armitage R, Arnedt JT. An Open Pilot of cognitive-behavioral therapy for Insomnia in Women with Postpartum Depression. Behav Sleep Med. 2012;11:1–11. 10.1080/15402002.2012.683902 23216373

[39] Owais S, Chow SHT, Furtado M, Frey BN, Van Lieshout RJ. Non-pharmacological interventions for improving postpartum maternal sleep: a systematic review and meta-analysis. Sleep Med Rev. 2018;41:87–100. 29449122 10.1016/j.smrv.2018.01.005

