# Supplementary material for: Longitudinal assessment of sleep and fatigue according to baby feeding method in postpartum women: a prospective observational study
Source: BMC Pregnancy Childbirth. 2024 Aug 12;24:529. doi: 10.1186/s12884-024-06671-0 (PMC11321152; doi:10.1186/s12884-024-06671-0)
Supplement: Supplementary file 1 — Supplementary Material 1 [file 12884_2024_6671_MOESM1_ESM.docx]

| **Table Appendix 1** | |  |  |  |  |  |  |
| --- | --- | --- | --- | --- | --- | --- | --- |
| Characteristics of participants according to feeding method at T1, two weeks postpartum. | | | | | | | |
|  |  | Total sample | Breastfeeding on T1 | Mixed feeding on T1 | Bottle feeding on T1 | Corr. | P |
|  |  |  |  |  |  |  |  |
|  |  | N= 163 | N= 102 | N= 28 | N= 33 |  |  |
| Age of mothers, mean ± SD, years | | 31.3 ± 4.5 | 31.1 ± 3.9 | 30.8 ± 6.3 | 32.1 ± 4.2 | .025 | .589 |
| Education | |  |  |  |  | -.085 | .140 |
|  | Primary school, n (%) | 2 (1.2%) | 0 (0%) | 2 (7.1%) | 0 (0%) |  |  |
|  | Secondary school, n(%) | 34 (20.9%) | 18(17.6%) | 9 (32.1%) | 7 (21.2%) |  |  |
|  | Professional degree, n(%) | 60 (36.8%) | 38 (37.3%) | 7 (25%) | 15 (45.5%) |  |  |
|  | Academic degree, n(%) | 12 (7.4%) | 8 (7.8%) | 2 (7.1%) | 2 (6.1%) |  |  |
|  | Master's degree, n(%) | 48 (29.4%) | 34 (33.3%) | 8 (28.6%) | 6 (18.2%) |  |  |
|  | PhD, n(%) | 7 (4.3%) | 4 (3.9%) | 0 (0%) | 3 (9.1%) |  |  |
| Marital status | |  |  |  |  | .001 | .986 |
|  | Single, n(%) | 4 (2.5%) | 0 (0%) | 3 (10.7%) | 1 (3.0%) |  |  |
|  | Partnered, n(%) | 77 (47.2%) | 51 (50%) | 13 (46.4%) | 13 (39.4%) |  |  |
|  | Married, n(%) | 82 (50.3%) | 51 (50%) | 12 (42.9%) | 19 (57.6%) |  |  |
| Work status | |  |  |  |  | -.010 | .107 |
|  | Unemployed, n(%) | 11 (6.7%) | 5 (4.9%) | 4 (14.3%) | 2 (6.1%) |  |  |
|  | Worker, n(%) | 8 (4.9%) | 3 (2.9%) | 2 (7.1%) | 3 (9.1%) |  |  |
|  | Employee, n(%) | 81 (49.7%) | 52 (51.0%) | 13 (46.4%) | 16 (48.5%) |  |  |
|  | Independent, n(%) | 19 (11.7%) | 10 (9.8%) | 3 (10.7%) | 6 (18.2%) |  |  |
|  | Civil servant, n(%) | 41 (25.2%) | 30 (29.4%) | 5 (17.9%) | 6 (18.2%) |  |  |
|  | Student, n(%) | 3 (1.8%) | 2 (2.0%) | 1 (3.6%) | 0 (0%) |  |  |
| **Employment rate (%), mean ± SD** | | **86.3 ± 28.5** | **89.9 ± 25.1** | **75.3 ± 37.7** | **84.4 ± 28.0** | **-.168*** | **.031** |
| Presence of disease at T0 | |  |  |  |  |  |  |
|  | Sleep disorder, n (%) | 0 (0%) | 0 (0%) | 0 (0%) | 0 (0%) |  |  |
|  | Obstructive Sleep Apnea, n(%) | 0 (0%) | 0 (0%) | 0 (0%) | 0 (0%) |  |  |
|  | Restless Legs Syndrome, n(%) | 4 (2.5%) | 2 (2%) | 1 (3.6%) | 1 (3%) | -.121 | .647 |
|  | **Anemia, n(%)** | **6 (3.7%)** | **6 (5.9%)** | **0 (0%)** | **0 (0%)** | **.389*** | **.012** |
|  | Hypothyroidism, n(%) | 7 (4.3%) | 4 (3.9%) | 1 (3.6%) | 2 (6.1%) | -.078 | .706 |
|  | Psychic disorder, n(%) | 3 (1.8%) | 1 (1.0%) | 1 (3.6%) | 1 (3.0%) | -.288 | .380 |
|  | Chronic Fatigue Syndrome, n(%) | 0 (0%) | 0 (0%) | 0 (0%) | 0 (0%) |  |  |
|  | Other disease, n(%) | 14 (8.6%) | 9 (8.8%) | 4 (14.3%) | 1 (3.0%) | .069 | .577 |
| **Mean number of hours sleep before pregnancy, mean ± SD** | |  |  |  |  |  |  |
|  |  | **7.80 ± 0.88** | **7.72 ± 0.88** | **7.91 ± 0.92** | **7.94 ± 0.85** | **.118*** | **.037** |
| Taking medication at T0, n(%) | | 43 (26.4%) | 22 (21.6%) | 14 (50%) | 7 (21.2%) | -.097 | .268 |
| T0: 35-37 weeks pregnancy; T1: two weeks postpartum. | | | | | | | |
| SD: standard deviation; Corr.: Somers'd ordinal correlation, | | | | | | | |
| ** p < .01, * p <.05 | |  |  |  |  |  |  |
