# Supplementary material for: Longitudinal assessment of sleep and fatigue according to baby feeding method in postpartum women: a prospective observational study
Source: BMC Pregnancy Childbirth. 2024 Aug 12;24:529. doi: 10.1186/s12884-024-06671-0 (PMC11321152; doi:10.1186/s12884-024-06671-0)
Supplement: Supplementary file 2 — Supplementary Material 2 [file 12884_2024_6671_MOESM2_ESM.docx]

| **Table Appendix 3** |  |  |  |  |  |  |
| --- | --- | --- | --- | --- | --- | --- |
| Descriptives of PSQI, ISI, FSS, CESD on T0, T1, T2 and their evolution between two moments for women who did not change their feeding method at T2. | | | | | | |
|  | Total sample | Breast feeding at T1 (C1=0) | Mixed feeding at T1 (C1 = 1) | Bottle feeding at T1 (C1 = 2) | Correl.^(^*^)^ with C1 (P value) | P value of test ^(^†^)^ of equal means between breast and bottle feeding |
|  |  |  |  |  |  |  |
|  | N = 115 | N= 77 | N = 11 | N = 27 |  |  |
| PSQI0: mean ± SD | 6.98 ± 3.02 | 6.74 ± 2.87 | 7.55 ± 2.42 | 7.44 ± 3.63 | .06 (.304) | .441 |
| PSQI1: mean ± SD | 7.43 ± 2.87 | 7.26 ± 2.87 | 7.64 ± 1.69 | 7.81 ± 3.28 | .04 (.454) | .414 |
| PSQI1 - PSQI0: mean ± SD | 0.45 ± 4.01 | 0.52 ± 3.37 | 0.09 ± 3.67 | 0.37 ± 5.64 | .02 (.798) | .792 |
| ISI0: mean ± SD | 8.77 ± 4.26 | 8.55 ± 4.41 | 8.82 ± 3.22 | 9.37 ± 4.26 | .06 (.264) | .26 |
| ISI1: mean ± SD | 7.98 ± 4.46 | 7.95 ± 4.74 | 7.64 ± 2.58 | 8.22 ± 4.32 | .02 (706) | .684 |
| ISI1 - ISI0: mean ± SD | -0.78 ± 5.41 | -0.60 ± 5.57 | -1.18 ± 2.96 | -1.15 ± 5.86 | -.02 (.622) | .741 |
| **FSS0: mean ± SD** | **3.75 ± 1.17** | **3.58 ± 1.19** | **3.93 ± 0.97** | **4.16 ± 1.11** | **.12 ^‡^ (.025)** | **.029 ^‡^** |
| FSS1: mean ± SD | 3.69 ± 1.16 | 3.62 ± 1.21 | 3.89 ± 1.04 | 3.78 ± 1.09 | .04 (.486) | .564 |
| FSS1 - FSS0: mean ± SD | -0.06 ± 1.17 | 0.04 ± 1.21 | -0.03 ± 1.07 | -0.38 ± 1.04 | -.12 (.093) | .110 |
| CESD0: mean ± SD | 18.11 ± 4.67 | 17.77 ± 4.88 | 20.64 ± 3.98 | 18.07 ± 4.13 | .05 (.349) | .774 |
| CESD1: mean ± SD | 17.78 ± 5.30 | 17.42 ± 5.54 | 19.36 ± 2.94 | 18.19 ± 5.34 | .05 (.354) | .623 |
| CESD1 - CESD0: mean ± SD | -0.33 ± 5.39 | -0.35 ± 5.81 | -1.27 ± 4.34 | 0.11 ± 5.38 | -.00 (.979) | .711 |
|  | Total sample | Breast feeding at T2 (C2=0) | Mixed feeding at T2 (C2 = 1) | Bottle feeding at T2 (C2 = 2) | Correl.^(^*^)^ with C2 (P value) | P value of test ^(^†^)^ of equal means between breast and bottle feeding |
|  |  |  |  |  |  |  |
|  | N = 115 | N = 77 | N = 11 | N = 27 |  |  |
| PSQI2: mean ± SD | 6.08 ± 3.31 | 6.32 ± 3.49 | 6.64 ± 1.69 | 5.15 ± 2.70 | -.10 (.165) | .123 |
| **PSQI2 - PSQI1: mean ± SD** | **-1.35 ± 3.04** | **-0.94 ± 2.91** | **-1.00 ± 2.45** | **-2.67 ± 3.33** | **-.16** **^‡^ (.030)** | **.01 ^‡^** |
| ISI2: mean ± SD | 6.76 ± 4.77 | 6.96 ± 5.36 | 7.36 ± 2.69 | 5.92 ± 3.41 | -.01 (.886) | .672 |
| ISI2 - ISI1: mean ± SD | -1.26 ± 4.25 | -0.99 ± 4.54 | -0.27 ± 3.29 | -2.50 ± 3.49 | -.08 (.253) | .174 |
| FSS2: mean ± SD | 3.40 ± 1.27 | 3.33 ± 1.35 | 3.78 ± 1.10 | 3.47 ± 1.12 | .06 (.400) | .641 |
| FSS2 - FSS1: mean ± SD | - 0.29 ± 0.95 | -0.27 ± 0.96 | -0.33 ± 0.84 | -0.31 ± 1.01 | -.04 (.626) | .642 |
| CESD2: mean ± SD | 18.04 ± 7.96 | 17.74 ± 7.46 | 19.09 ± 7.06 | 18.50 ± 9.82 | .00 (.962) | .910 |
| CESD2 - CESD1: mean ± SD | 0.25 ± 7.57 | -0.32 ± 6.95 | -0.27 ± 7.86 | 0.27 ± 9.34 | -.02 (.814) | .812 |
| ^(^*^)^ Somers' d ordinal correlation  ^(^†^)^ T test (in the cases where the two samples are normally distributed according to the Shapiro-Wilk test of normality) or Mann Whitney test (in the other cases)  Measurement moments: T0, 35 weeks pregnancy; T1, 2 weeks postpartum; T2, 8 weeks postpartum.  PSQI0, PSQI1, PSQI2: PSQI on moments T0 respectively T1, T2 and similarly for ISI, FSS, CESD  ‡ p < .05; ** p < .01,  SD: Standard deviation | | | | | | |
