# Supplementary material for: Longitudinal assessment of sleep and fatigue according to baby feeding method in postpartum women: a prospective observational study
Source: BMC Pregnancy Childbirth. 2024 Aug 12;24:529. doi: 10.1186/s12884-024-06671-0 (PMC11321152; doi:10.1186/s12884-024-06671-0)
Supplement: Supplementary file 3 — Supplementary Material 3 [file 12884_2024_6671_MOESM3_ESM.docx]

| **Table Appendix 4** |  | |  | |
| --- | --- | --- | --- | --- |
| Descriptives of PSQI, ISI, FSS, CESD on T0, T1, T2 and their evolution between two moments for women who changed their feeding method at T2 (N = 26) | | | | |
| PSQI0: mean ± SD | | 7.38 ± 3.25 | |  |
| PSQI1: mean ± SD | | 8.73 ± 3.31 | |  |
| PSQI1 - PSQI0: mean ± SD | | 1.35 ± 3.65 | |  |
| ISI0: mean ± SD | | 8.69 ± 4.75 | |  |
| ISI1: mean ± SD | | 9.96 ± 5.17 | |  |
| ISI1 - ISI0: mean ± SD | | 1.27 ± 5.57 | |  |
| FSS0: mean ± SD | | 3.79 ± 1.23 | |  |
| FSS1: mean ± SD | | 3.95 ± 1.36 | |  |
| FSS1 - FSS0: mean ± SD | | 0.17 ± 1.30 | |  |
| CESD0: mean ± SD | | 18.96 ± 5.46 | |  |
| CESD1: mean ± SD | | 22.00 ± 8.03 | |  |
| CESD1 - CESD0: mean ± SD | | 3.04 ± 8.64 | |  |
| PSQI2: mean ± SD | | 7.85 ± 2.94 | |  |
| PSQI2 - PSQI1: mean ± SD | | -0.88 ± 3.23 | |  |
| ISI2: mean ± SD | | 9.50 ± 5.69 | |  |
| ISI2 - ISI1: mean ± SD | | -0.46 ± 3.94 | |  |
| FSS2: mean ± SD | | 3.96 ± 1.45 | |  |
| FSS2 - FSS1: mean ± SD | | 0.01 ± 1.15 | |  |
| CESD2: mean ± SD | | 20.50 ± 8.13 | |  |
| CESD2 - CESD1: mean ± SD | | -1.50 ± 6.33 | |  |
| SD: Standard deviation PSQI: Pittsburgh Sleep Quality Index; ISI: Insomnia Severity Index; FSS: Fatigue Severity Scale; CESD: Center for Epidemiologic Studies Depression Scale | | | | |
