# Supplementary material for: Longitudinal assessment of sleep and fatigue according to baby feeding method in postpartum women: a prospective observational study
Source: BMC Pregnancy Childbirth. 2024 Aug 12;24:529. doi: 10.1186/s12884-024-06671-0 (PMC11321152; doi:10.1186/s12884-024-06671-0)
Supplement: Supplementary file 4 — Supplementary Material 4 [file 12884_2024_6671_MOESM4_ESM.docx]

| **Table Appendix 2** | | | | | | | |
| --- | --- | --- | --- | --- | --- | --- | --- |
| Associations of the decision, on T2, to change the feeding method (mothers giving breastfeeding or mixed feeding on T1), with parameters on T1 | | | | | | | |
|  |  |  |  |  |  |  |  |
| 1. Somers'd correlations between the parameters on T1 and the dichotomous variable Ch2, the decision to change the feeding method on T2 (Ch2 dependent, values 0, nochange, and 1, change). | | | | | | | |
|  | PSQI1 | ISI1 | FSS1 | CESD1 |  |  |  |
| Value | .109 | .090 | .042 | .112 |  |  |  |
| Standard Error | .054 | .049 | .049 | .048 |  |  |  |
| Sig. | .043* | .065 | .369 | .021* |  |  |  |
|  |  |  |  |  |  |  |  |
|  | | | | | | | |
| 2. Backwards (LR) Logistic Regression with Ch2 dependent and PSQI1, ISI1, CESD1 and PSQI1 as independent variables. Only CESD1 was retained in the final model. | | | | | | | |
|  |  |  |  |  |  |  |  |
|  | Estimate | St. Err. | Wald | df | Sig. |  |  |
| Constant | -3.317 | .794 | 17.457 | 1 | .000 |  |  |
| CESD1 | .107 | .038 | 8.135 | 1 | .004 ^†^ |  |  |
| Model fit: Chi-square = 9.176 (df = 1), sig.= .002† Pseudo Rsquare = .075 (Mc Fadden, R²L); .077 (Cox and Snell); .117 (Nagelkerke) PSQI: Pittsburgh Sleep Quality Index; ISI: Insomnia Severity Index; CESD: Center for Epidemiologic Studies Depression Scale | | | | | | | |
|  |  |  |  |  |  |  |  |
| * p<.05; † p<.01 | |  |  |  |  |  |  |
